# The elusive diagnosis and emergent management of a late-presenting ruptured interstitial pregnancy: a case report

**DOI:** 10.1186/s12884-021-04026-7

**Published:** 2021-08-13

**Authors:** Lauren M. Ahlschlager, David Mysona, A. Jenna Beckham

**Affiliations:** 1 University of North Carolina at Chapel Hill School of Medicine, 321 S Columbia St, Chapel Hill, NC 27516 USA; 2 Department of Obstetrics and Gynecology, University of North Carolina School of Medicine, Chapel Hill, USA; 3 Department of Obstetrics and Gynecology, WakeMed Health and Hospitals, 3024 New Bern Ave Suite 309, Raleigh, NC 27610 USA

**Keywords:** Interstitial pregnancy, Ectopic, Ultrasound, Obstetrics, Case report

## Abstract

**Background:**

Interstitial pregnancies are rare and often difficult to diagnose given their proximal position to the uterine cavity, however most are identified by 12 weeks gestation. Delayed or missed diagnosis contributes to heightened incidence of poor outcomes including hemorrhage and death.

**Case presentation:**

A 35-year-old woman at 15 weeks gestation with confirmed intrauterine pregnancy on first trimester ultrasound and prior negative MRI presented in hemorrhagic shock and was found to have a ruptured interstitial pregnancy. Exploratory laparotomy revealed the fetus to be in the abdomen as well as a large cornual defect and abnormal placentation that resulted in supracervical hysterectomy.

**Conclusions:**

Interstitial pregnancy should be considered in a patient presenting with symptoms consistent with ectopic rupture, especially in the setting of equivocal or suboptimal prior imaging. Earlier diagnosis may allow for fertility-sparing intervention and decreased risk of morbidity and mortality.

## Teaching points

1. First trimester dating ultrasounds and even advanced imaging such as MRI have difficulty visualizing and definitively diagnosing interstitial ectopic pregnancies.
2. Clinical suspicion for an interstitial ectopic pregnancy should remain high if a patient has persistent symptoms consistent with an ectopic pregnancy despite prior imaging indicating an intrauterine pregnancy.
3. While fertility-preserving and conservative surgical approaches are the ideal management for an interstitial ectopic pregnancy, in certain cases hysterectomy may be necessary.

## Background

Ectopic pregnancies account for 2 % of all pregnancies but are the cause of nearly 3 % of maternal deaths in the United States [1]. While interstitial pregnancies make up 2 to 4 % of ectopic pregnancies, they are associated with significant mortality, accounting for 20 % of all deaths associated with ectopic pregnancies [2, 3].

Interstitial, cornual, and angular pregnancy are terms that are often used interchangeably, likely secondary to close anatomic proximity. However, each of these has a specific definition. A cornual pregnancy is most accurately defined as one occurring “in the rudimentary horn of a uterus with a Mullerian anomaly,” though many practitioners use this term to describe any pregnancy occurring near the cornua [3]. Angular pregnancies are classified as occurring within the uterine cavity but just medial to the uterotubal junction. Interstitial pregnancies, by contrast, are gestations occurring in the most proximal portion of the fallopian tube, embedded in myometrium, and thus are considered extrauterine because they are not within the uterine cavity [3].

Differentiation between an interstitial ectopic versus an eccentrically located intrauterine pregnancy (cornual or angular) is challenging and often leads to delayed diagnosis of interstitial ectopic pregnancies, contributing to a higher mortality rate for these pregnancies [4]. Such distinctions are exceedingly important as management of these conditions varies greatly. Whereas eccentrically located intrauterine pregnancies often result in healthy term pregnancies, interstitial ectopic pregnancies are rarely viable, with a rupture rate of approximately 15 % [3].

Here we present the unique case of a woman in hemorrhagic shock who was found to have an undiagnosed interstitial ectopic pregnancy at 15 weeks despite prior first trimester ultrasound and recent magnetic resonance imaging (MRI) which were interpreted as indicating a normal intrauterine pregnancy. This case underscores the importance of both early detection and high suspicion for interstitial ectopic pregnancies even in the setting of negative prior imaging.

## Case presentation

A 35-year-old G3P2002 with two prior uncomplicated vaginal deliveries and no significant medical history presented at 15 3/7 weeks gestation by last menstrual period. She was brought in by emergency medical services due to a syncopal episode at home. Her history was notable for one-month of progressive abdominal pain and associated nausea, vomiting and diarrhea. She reported having regular follow-up with her obstetrician and multiple recent emergency department visits. She had no prior surgeries. Her vitals showed a heart rate in the 130s and her blood pressure was 76/43. On physical exam her abdomen was diffusely tender with peritoneal signs. Her labs were significant for a leukocytosis to 28,700, hemoglobin of 7.6, creatinine of 1.07 mg/dL and lactate of 5.7 mmol/L. Ultrasonography showed free fluid in the abdomen. Both the obstetrics and trauma surgery teams were immediately consulted as the source of her hemorrhagic shock was unknown. The patient became hemodynamically stable with resuscitation; so, the decision was made to proceed with a computed tomography (CT) scan of the abdomen and pelvis with contrast.

**Fig. 1:**
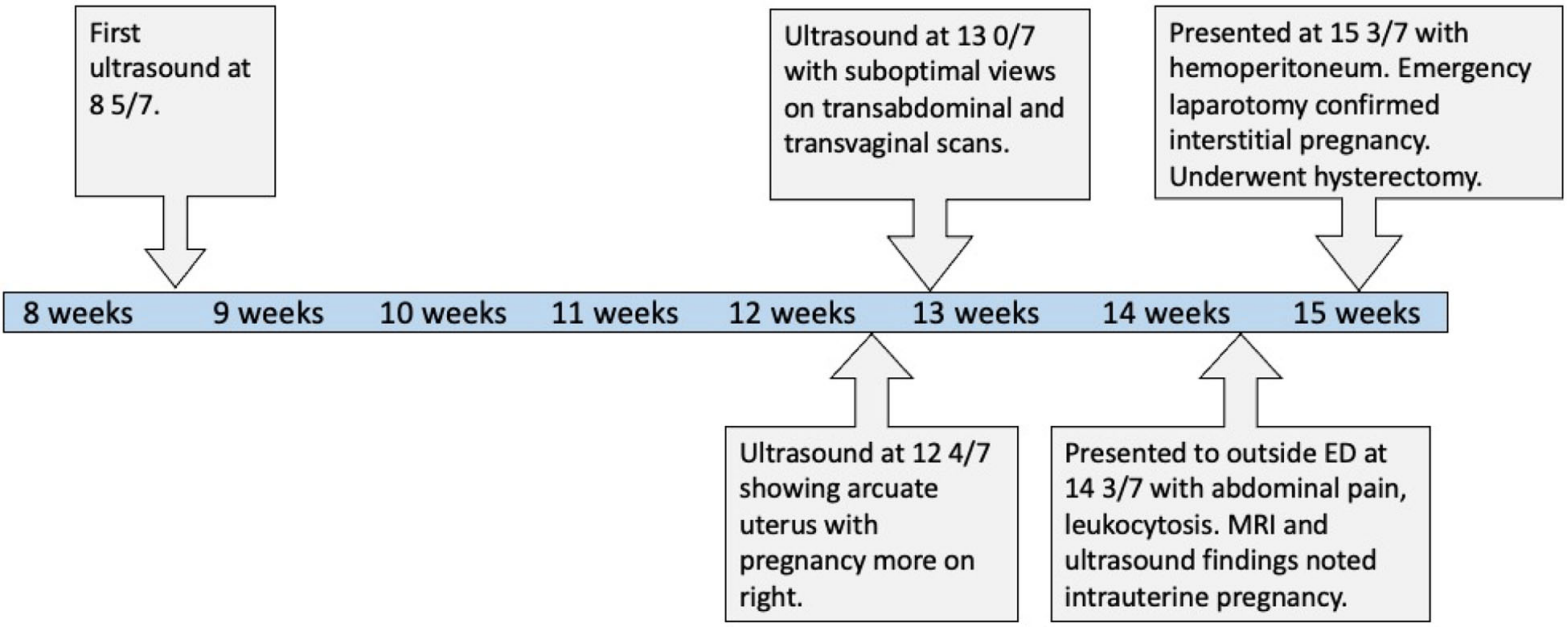
Timeline of events

Concurrently, outside hospital records were made available and reviewed by both teams. They revealed that the patient had ultrasounds performed at 8 5/7, 12 4/7, and 13 0/7. Her ultrasound at 12 4/7 noted a possible arcuate uterus with the pregnancy located more on the patient’s right. An additional scan at 13 0/7 reported suboptimal visualization of the pregnancy on both transabdominal scan and transvaginal scan. These records also showed that one week prior to presentation at our facility, the patient had gone to an outside emergency department with right lower quadrant pain. There she had an elevated white blood cell count to 18,000, a transabdominal ultrasound and abdominal MRI. Neither the ultrasound nor the MRI were interpreted as showing any acute abnormalities. Both specifically commented on there being an intrauterine pregnancy. The patient was discharged home from the outside emergency department with a presumed viral illness. A timeline of events is presented in Fig. 1.

**Fig. 2:**
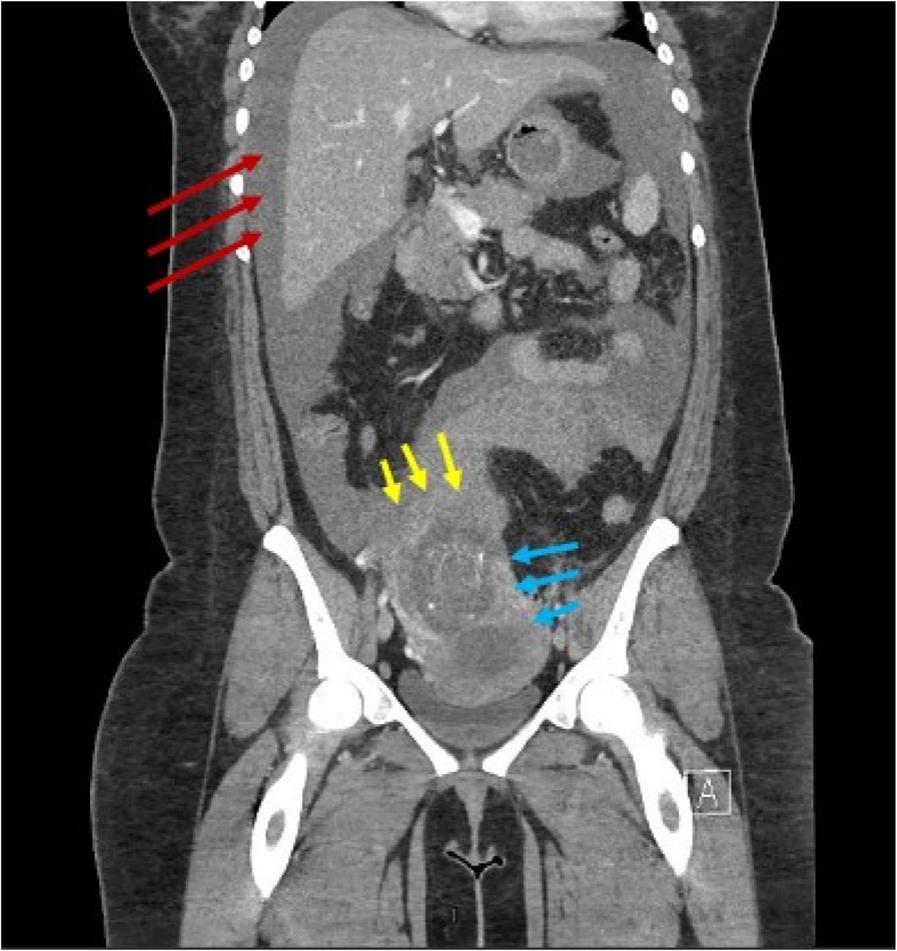
CT scan of the abdomen and pelvis in the coronal plane. The scan revealed large volume hemoperitoneum (red arrows), location of interstitial ectopic pregnancy (blue arrows), an empty uterine cavity (blue arrows), and the area of suspected rupture (yellow arrows)

At our facility the patient’s CT scan demonstrated a large volume hemoperitoneum, and the pregnancy appeared to be superior to the uterine corpus. This raised concern for a ruptured interstitial ectopic pregnancy (Fig. 2). The patient was consented for emergency laparotomy with possible hysterectomy.

**Fig. 3:**
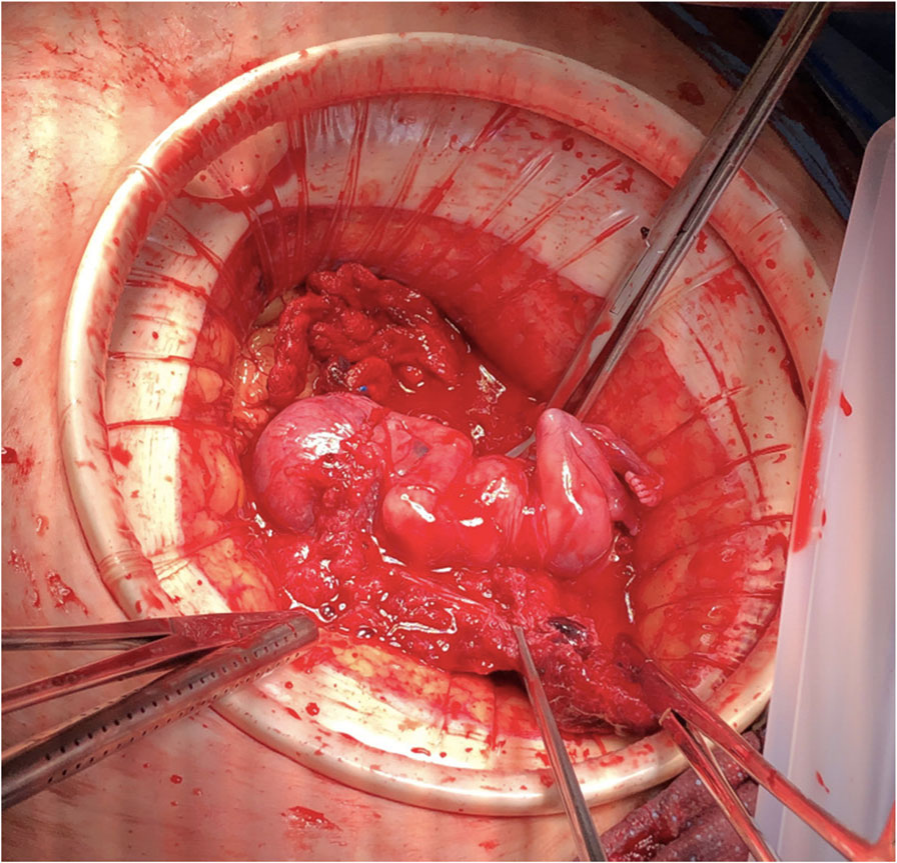
Intraoperative view of fetus as encountered in abdomen

**Fig. 4:**
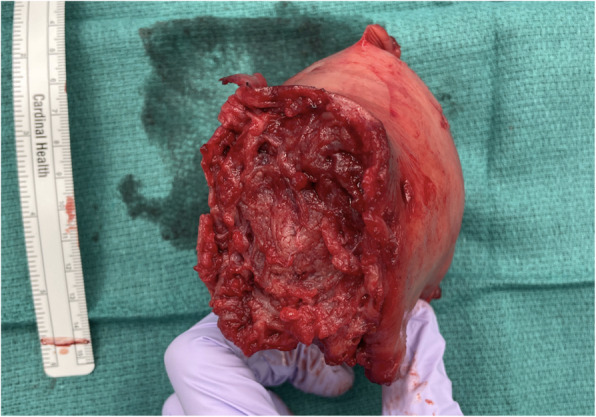
Large cornual defect resulting from rupture of the interstitial ectopic pregnancy

A midline laparotomy incision was made below the umbilicus. Upon entering the abdominal cavity, hemoperitoneum was encountered and more than 2 L of blood were evacuated from the abdomen. Inspection of the uterus showed an enlarged right cornua with the amniotic sac actively rupturing through the uterine serosa. Gentle manipulation of the uterus resulted in spontaneous rupture of the amniotic sac and expulsion of the pregnancy (Fig. 3). The patient was hemodynamically stable as resuscitation was ongoing. Further inspection of the operative field showed brisk bleeding from placental tissue densely adherent to the uterus and what remained of the right uterine cornua. On close examination, there was no remaining tissue in the right cornua which could be used for re-approximation and no clear plane for dissection or removal of the remaining placental tissue (Fig. 4). Thus, the decision was made to proceed with supracervical hysterectomy which was uncomplicated. Intraoperative blood loss was 5000 mL. The patient received 6000 mL intravenous fluid, 6 units packed red blood cells, 4 units fresh frozen plasma, 1 unit platelets and 500 mL albumin. Preoperative hemoglobin was 7.6 g/dL and was 8.4 g/dL on postoperative day 1. The surgical pathology report was significant for right cornual changes consistent with placenta percreta.

Her postoperative course was complicated by the need for brief use of supplemental oxygen on postoperative night 0. This was thought to be due to atelectasis versus volume overload secondary to large volume resuscitation. The patient and her spouse received emotional support and counseling services while in the hospital and the patient was started on sertraline. She was discharged on postoperative day 3 with plans for a two-week follow-up. At her two-week follow-up the patient had recovered from a surgical perspective. However, emotionally, the patient had developed significant anxiety as a result of the prior events.

## Discussion and conclusions

Ruptured interstitial ectopic pregnancies are a rare but potentially fatal occurrence with mortality rates 7 times higher than that of other ectopic pregnancies. The increased risk of maternal mortality in interstitial pregnancies can be partially attributed to difficulty in diagnosis which often results in missed or delayed identification [5]. Diagnostic criteria for interstitial ectopic pregnancy on ultrasound include (1) an empty uterine cavity (2) a gestational sac located at least 1 cm from lateral uterine wall, and (3) a thin (< 5mm) myometrial layer surrounding the gestational sac. Presence of an “interstitial line sign” has also been posed as an additional diagnostic criterion and refers to visualization of a thin, echogenic line on ultrasound representing the interstitial region of the fallopian tube just lateral to the gestational sac and endometrial cavity [3, 6]. Notably, these criteria are only valid in the first trimester before the gestational sac enlarges. In the case of our patient, her first trimester ultrasound at 8 5/7 weeks reportedly did not support the diagnosis [7].

**Fig. 5:**
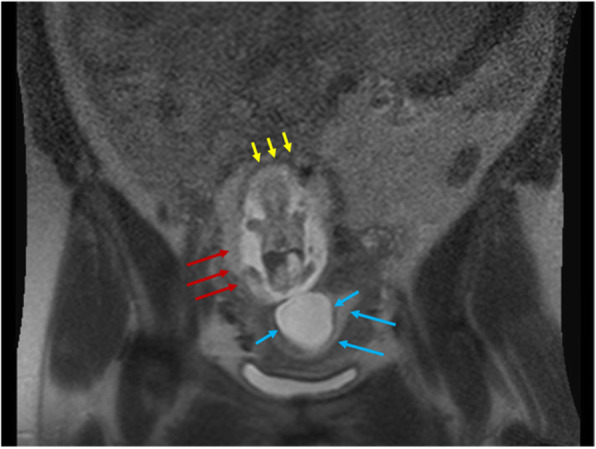
MRI demonstrating the gestational sac enveloped in myometrium (red arrows). The amniotic sac is hour glassing into the endometrial cavity and uterine decidua appears to be located adjacent to gestational sac (blue arrows). A thin layer of myometrium seems to be intact and surrounding the interstitial pregnancy (yellow arrows)

Compared to ultrasound, considerably less has been documented regarding diagnostic MRI findings associated with interstitial pregnancies. MRI may be an important tool particularly in cases of suspected second trimester rupture when ultrasound findings become more equivocal. Presence of an eccentrically located gestational sac embedded in a thin, asymmetric layer of myometrium with visualization of uterine decidua adjacent to the sac are features of second-trimester interstitial pregnancy [7]. On retrospective review of our patient’s MRI, all of these criteria seem to be met (Fig. 5).

Unfortunately, due to the difficulty in diagnosing interstitial ectopic pregnancies they are associated with increased morbidity and mortality compared to ectopic pregnancies of tubal origin. In ruptured interstitial pregnancies, the rate of hysterectomy has been reported as high as 40 %. The mortality rate of any interstitial pregnancy has been estimated between 2 and 2.5 % which is 7 times higher than average for ectopic pregnancies [6]. The high morbidity and potential mortality associated with interstitial ectopic may be attributable in part to the high degree of vascularization of the utero-tubal region from branches of the ovarian and uterine arteries. It has traditionally been posited that the interstitial region of the tube is more distensible compared to more distal tubal segments. Therefore, the interstitial region can accommodate pregnancies of later gestational ages before rupturing. Evidence has shown rupture of interstitial ectopic pregnancies commonly occurs prior to 12 weeks [8]. The patient presenting at 15 weeks with a ruptured interstitial ectopic pregnancy represents both a rare event and one with an especially high risk of life-threatening hemorrhage.

Surgical options for management of interstitial ectopic pregnancies include salpingostomy, cornuostomy, and cornual resection. These techniques have been commonly reported utilizing laparotomy. However, minimally invasive laparoscopic techniques are becoming more routine. Laparotomy with hysterectomy is generally reserved for cases of hemodynamic instability [5].

Our hypothesis is that this patient’s pregnancy originated as a true interstitial ectopic pregnancy, which was initially missed on first trimester ultrasound. As the pregnancy continued to grow, it subsequently extended beyond the interstitial myometrium and into the adjacent endometrium. This likely led to the appearance of an “arcuate uterus” mentioned on the prior ultrasound report. Ultimately, the pregnancy outgrew the cornua and began to erode through the uterine serosa leading to her multiple presentations and eventual uterine rupture. As it relates to the findings of placenta percreta on the pathology report, we believe that due to the abnormal site of implantation in combination with the demands placed on the placenta to support the pregnancy, the placenta likely eroded through the myometrial wall further contributing to the eventual rupture of the pregnancy.

This report is limited by access to only outside facility interpretations of the patient’s initial ultrasounds rather than original images that may have provided additional findings on retrospective review. At the same time this case report benefits from the illustrative examples of MRI and CT images that support the ultimate diagnosis.

In conclusion, the negative readings of the ultrasound and MRI imaging performed prior to this patient’s presentation highlight the difficult nature of diagnosing interstitial ectopic pregnancies. This case emphasizes the importance of maintaining a high index of suspicion in a patient with unresolving symptoms. This patient’s late presentation at rupture led to a large cornual defect and profound hemorrhage which fortunately did not end in maternal mortality. Earlier diagnosis may have allowed for a more conservative, fertility-preserving intervention such as methotrexate therapy or uterine artery embolization. We report this case in hopes that it will encourage consideration of this rare but potentially deadly condition in patients who present with symptoms suggestive of ectopic pregnancy in the setting of suboptimal or equivocal prior imaging, as keeping a broad differential diagnosis may reduce mortality and improve patient outcomes.

## Data Availability

All data analyzed during this study are included herein.

## Abbreviations

CT: Computed tomography
MRI: Magnetic resonance imaging
